# Functional properties of human platelets derived *in vitro* from CD34^+^ cells

**DOI:** 10.1038/s41598-020-57754-9

**Published:** 2020-01-22

**Authors:** V. Do Sacramento, L. Mallo, M. Freund, A. Eckly, B. Hechler, P. Mangin, F. Lanza, C. Gachet, C. Strassel

**Affiliations:** Université de Strasbourg, INSERM, EFS-Grand Est, BPPS UMR_S1225, FMTS, F-67000 Strasbourg, France

**Keywords:** Biotechnology, Medical research

## Abstract

The *in vitro* production of blood platelets for transfusion purposes is an important goal in the context of a sustained demand for controlled products free of infectious, immune and inflammatory risks. The aim of this study was to characterize human platelets derived from CD34^+^ progenitors and to evaluate their hemostatic properties. These cultured platelets exhibited a typical discoid morphology despite an enlarged size and expressed normal levels of the major surface glycoproteins. They aggregated in response to ADP and a thrombin receptor agonist peptide (TRAP). After infusion into NSG mice, cultured and native platelets circulated with a similar 24 h half-life. Notably, the level of circulating cultured platelets remained constant during the first two hours following infusion. During this period of time their size decreased to reach normal values, probably due to their remodeling in the pulmonary circulation, as evidenced by the presence of numerous twisted platelet elements in the lungs. Finally, cultured platelets were capable of limiting blood loss in a bleeding assay performed in thrombocytopenic mice. In conclusion, we show here that cultured platelets derived from human CD34^+^ cells display the properties required for use in transfusion, opening the way to clinical trials.

## Introduction

Although the *in vitro* production of transfusion grade human platelets is a goal pursued by several laboratories worldwide, a number of obstacles still remain to be surmounted before we may consider these platelets as a real transfusion alternative. The reported low yields of 50–200 platelets per megakaryocyte (MK) and expected high production costs cast doubts on the prospect of transfusing cultured platelets on a large scale. A reasonable application might nevertheless be envisaged for well-identified needs, as in cases of transfusion failure due to platelet alloimmunization or the development of refractoriness^1,2^.

In this objective, efforts to improve different aspects of *in vitro* platelet production are well warranted. These include the selection of optimal progenitors to achieve adequate cell expansion without hampering differentiation^3,4^, the optimization of culture procedures to reach the highest possible level of MK maturation closely matching that observed in the bone marrow and a major improvement in the platelet release of these mature MK by mimicking native flow and environmental conditions^5–9^. Recently, a significant step forward was accomplished by the group of K. Eto, who reported the production of large numbers of platelets from iPSC-derived immortalized MK in a bioreactor with turbulent flow^10^.

In previous work, we observed that culture of CD34^+^ cells in the presence of SR1 led to the emergence of a CD34^+^CD41^low^ population with an increased capacity to generate platelet-like elements^11^. The aim of the present study was to determine whether these cultured platelets met the requirements for transfusion. In particular, we explored their capacity to recirculate and to ensure a hemostatic protection equivalent to that of donor-derived platelets.

## Materials and Methods

### MK differentiation in culture

CD34^+^-enriched cells from leukofilters (TACSI, Terumo BCT, Zaventem, Belgium) were expanded using a previously described two-phase optimized protocol^11^. Briefly, the filter extract was enriched in CD34^+^ cells by magnetic activated cell sorting (CD34 MicroBead Kit UltraPure, Miltenyi Biotec, Bergisch Gladbach, Germany). The cells were then seeded in StemSpan serum-free expansion medium (SFEM) supplemented with 20 µg/mL human low-density lipoprotein and a cocktail of cytokines (CC220, Stemcell Technologies, Vancouver, BC, Canada) and with 1 µM SR1 (Stemcell Technologies). On day 7, the cells were harvested, washed, seeded in StemSpan SFEM containing 1 µM SR1, 50 ng/mL TPO and 20 µg/mL human low-density lipoprotein and cultured for an additional 6 days. The cultures were incubated at 37 °C under normoxic conditions and a 5% CO_2_ atmosphere.

### Platelet isolation

Cultured Platelets were harvested after addition of 0.5 µM PGI_2_ and 0.02 U/mL apyrase to the culture plates followed by successive pipetting. The platelet-like particles were then centrifuged and resuspended in Tyrode’s albumin buffer as previously described^11^.

### Platelet ultrastructure and morphology

#### Transmission electron microscopy

Cultured platelets or native platelets were fixed in 2.5% glutaraldehyde and embedded in Epon. Thin sections were stained with uranyl acetate and lead citrate and examined under a JEol 2100-plus (Jeol, Japan)^12^.

#### Confocal microscopy

After fixation in paraformaldehyde, platelets were cytospun, permeabilized with 0.1% Triton X-100 in PBS and incubated sequentially for 30 min with an anti-ß1-tubulin mAb (1:400, 1 μg/mL, Eurogentec, Liège, Belgium) followed by a secondary GAM-488 antibody (10 µg/mL) and an anti-GPIIbIIIa mAb (Alma.17–647, 10 µg/mL) in PBS containing 1% BSA. The cells were then embedded in Mowiol (Mountant, Permafluor, Thermo Fisher Scientific, UK) and examined under a confocal microscope (TCS SP8, Leica Microsystems, Rueil-Malmaison, France) equipped with an oil objective (Type F immersion liquid, ne^23^ = 1,5180, ve = 46, Leica Microsystems). Data were acquired with LASAF software, version 1.62 (Leica Microsystems). Mouse lungs were embedded in a cryogenic gel and serial longitudinal cryosections were stained and observed as previously reported^13^.

### *In vitro* platelet studies

#### Platelet aggregation

Aggregation was measured at 37 °C by a standard turbidimetric method in an APACT 4004 aggregometer (ELITech Group, Puteaux, France)^14^. Briefly, a 135 μL aliquot of platelet suspension containing 20.10^6^. Cultured platelets or Native platelets was stirred at 1,100 rpm and activated by addition of 5 µM ADP in the presence of human fibrinogen (0.05 mg/mL), 10 µM TRAP or 0.1 U/mL thrombin, in a final volume of 150 µL. The extent of aggregation was estimated with APACT LPC software.

#### RNA content and GP expression

Thiazol orange labeling of RNA and surface expression of the major platelet glycoproteins (GP) were analyzed by flow cytometry as previously described^15,16^.

### *In vivo* functionality and hemostatic properties

#### Recirculation after infusion

Aliquots containing 1.10^8^ washed human Cultured or Native platelets were injected through the retro-orbital vein into macrophage-depleted (by clodronate liposome abdominal injection on day −1), 7 to 8 week-old female NSG (NOD.Cg-Prkdc scid, Il2rg tm1Wjl/SzJ) mice (Jackson laboratory, Bar habor, USA). Circulating human and mouse platelets were analyzed by flow cytometry in whole blood samples drawn 3, 6, 15, 30, 120, 240, 1400, 2800 and 4320 min after transfusion. Human platelets were detected with a mAb (ALMA.17) against human GPIIb-IIIa. A mAb against GPIbβ (RAM.1) which reacts with human and mouse platelets was used to delineate the platelet region on the plots^17^. The proportion of circulating Cultured or Native platelets recorded in the acquisition gate 3 min after transfusion was arbitrarily set to 1.

#### Bleeding assay

Aliquots containing 3.10^8^ washed human Cultured or Native platelets were injected retro‐orbitally into NSG mice made severely thrombocytopenic following intravenous administration of the rat anti-mouse GPIbα antibody (RAM.6, 5 mg/kg) 24 h prior to the bleeding assay. The tail bleeding time was measured 10 min after infusion of human platelets as described previously. The time required for the arrest of bleeding and the blood loss were recorded over 10 min^16,18^.

### Ethics statement

Human studies were performed according to Helsinki declaration. Control human samples were obtained from volonteer blood donors who gave written informed consent recruited by the blood transfusion center where the research was performed (Etablissement Français du Sang-Grand Est). All procedures were registered and approved by the French Ministry of Higher Education and Research and registered under the number AC_2015_2371.The donors gave their approval in the CODHECO number AC- 2008 - 562 consent form, in order for the samples to be used for research purposes. NSG mice were housed under pathogen-free conditions and all procedures were performed in accordance with the European Union Guideline 2010/63/EU. The study was approved by the Regional Ethical Committee for Animal Experimentation of Strasbourg, CREMEAS (CEEA 35) and registered under the number 10669.

### Statistical analyses

Results were expressed as the mean ± SEM and statistical comparisons were performed using an unpaired, two-tailed Student’s t-test or one-way ANOVA followed by the Bonferoni post-hoc test (Prism, Graph-Pad Software Inc., San Diego, CA, USA). P values of less than 0.05 were considered to be statistically significant.

## Results

### Morphological characterization of cultured platelets

**Figure 1:**
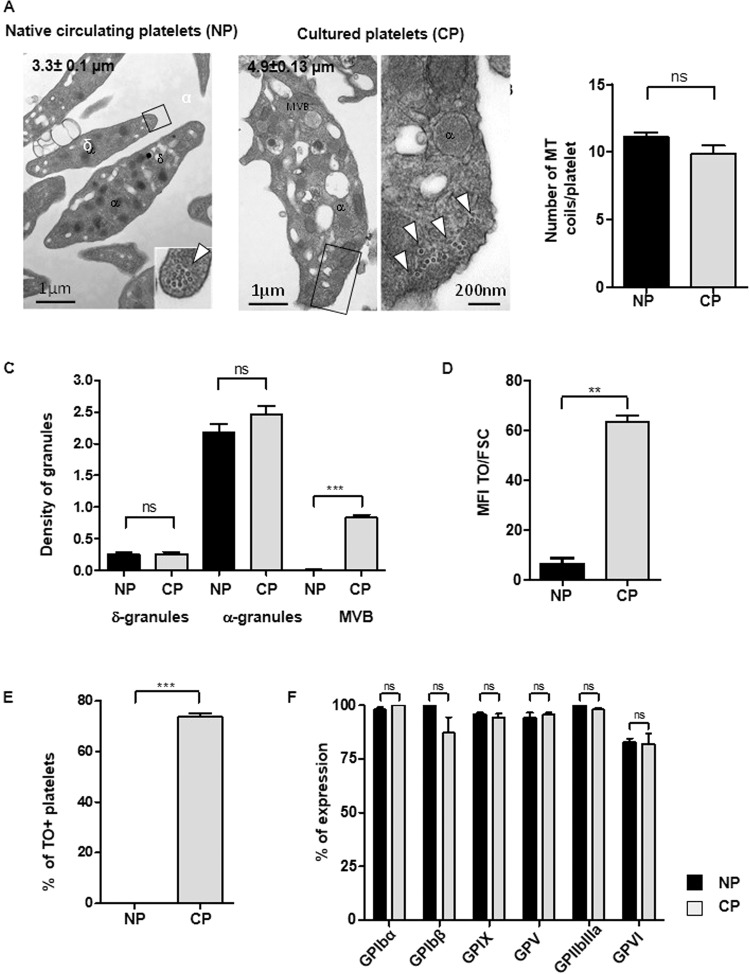
Characteristics of cultured platelets. (**A**) *Platelet ultrastructure*. Representative transmission electron microscopy (TEM) images of Native platelets (left panel, Bar = 1 µm) and Cultured platelets (right panel, Bar = 1 µm and 200 nm). **(B)** *Quantification of MT coils*. Numbers of microtubule coils in the marginal band on TEM images of thin sections of Native platelets and Cultured platelets. Values are the mean ± SEM for 40 and 82 platelets, n = 3, ns = not significant. **(C)** *Quantification of granules*. The densities of δ-granules, α-granules and multivesicular bodies (MVB) were quantified by TEM. Values are the density of granules reported to the platelet’s size ± SEM, ***p < 0.001, n = 70 and 95 for Native- and Cultured-Platelets respectively, n = 3. **(D,E)** *Reticulated platelets*. The bar graphs represent (**D**) the mean fluorescence intensity of thiazol orange (TO) staining in Native platelets or Cultured platelets reported to the platelet size represented by the forward scatter (FSC). (**E**) The bar graphs represent the percentage of platelets positive for TO and (**F**) the level of expression of the major surface glycoproteins.

Cultured platelets were first examined for their ultrastructure and degree of maturity. Cultured platelets were discoid and displayed a marginal band characteristic of Native platelets containing a similar number of microtubule coils (9.8 ± 0.6 Cultured Platelets, n = 40 vs 11.11. ± 0.3, Native platelets n = 82, ns; p > 0.05, n = 3). The diameter of Cultured platelets was however enlarged by 1.5 fold as compared to Native platelets (4.9 ± 0.13 vs 3.3 ± 0.1 µm, n = 70 and 95 respectively, n = 3) (Fig. 1A,B). They had normal densities per platelet section of α-granules (2.2 ± 0.1 for Native platelets vs 2.4 ± 0.1 for cultured platelets, n = 70 and 95 respectively, ns, n = 3) and δ-granules (0.3 ± 0.05 for Native platelets vs 0.2 ± 0.03 for cultured platelets, n = 70 and 95 respectively, ns, n = 3) (Fig. 1C). Cultured platelets nevertheless displayed signs of immaturity illustrated by an increased density of multivesicular bodies (MVB), which represent granules in formation (0.6 ± 0.01 Cultured Platelets vs 0.06 ± 0.01 Native platlets, n = 95 and 50 respectively, ***p < 0.001), and an enriched endoplasmic reticulum (Fig. 1A. C). These immature properties, classically observed in newly formed platelets, were confirmed by thiazol orange (TO) staining, with more than 90% of Cultured Platelets being TO positive as compared to less than 2% of Native platelets (Fig. 1D,E).

### Functional characteristics of cultured platelets

**Figure 2:**
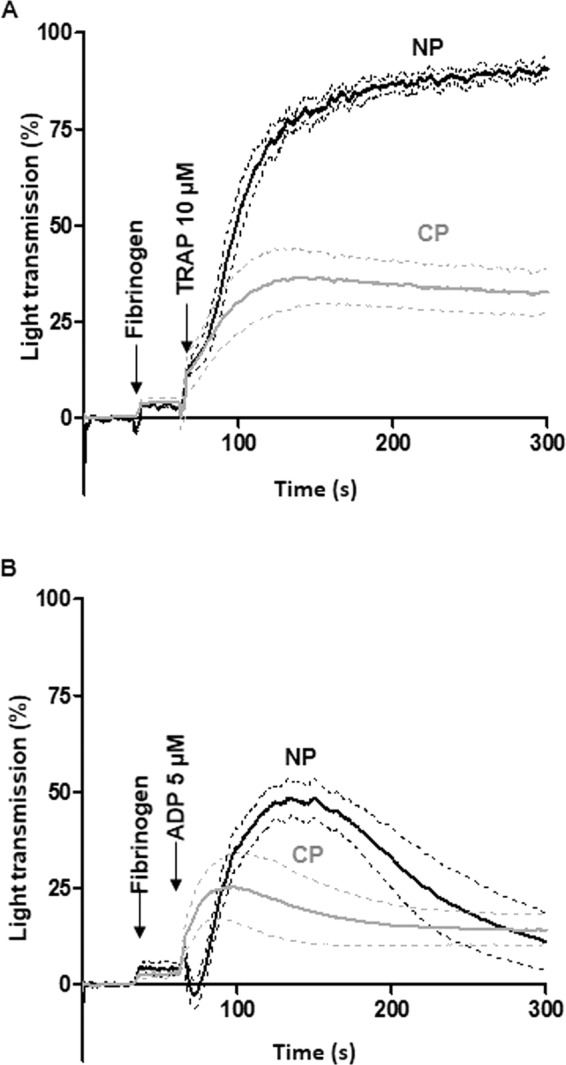
Reactivity of cultured platelets. (**A,B)** *Aggregation assay*. Washed platelets were stimulated with (**A**) TRAP (10 µM) (Cultured platelets vs Native platelets: 32.5 ± 5.8% vs 90.5 ± 1.9% increase in light transmission, n = 3) and (**B**) ADP (5 µM) (24.9 ± 7.9% vs 48.4 ± 4.6% increase in light transmission, n = 3) in the presence of fibrinogen. Tracings ± SEM (dotted lines).

We first established that there were no major differences between Cultured and Native platelets in the expression of the main platelet surface glycoproteins (Fig. 1F). The functionality of cultured platelets was then assessed *in vitro* using a standard turbidimetric aggregation assay. TRAP stimulation (10 µM) of Cultured Platelets resulted in 36 ± 7% of the maximal aggregation response of Native platelets (Fig. 2A). The responsiveness of Cultured Platelets was also demonstrated using ADP (5 µM), a weak agonist which enables one to easily detect functional defects. A rapid onset of aggregation was observed in Cultured Platelets suspensions, with a maximum representing up to 80% of that in Native platelets suspensions (58.6 ± 11.2%, n = 3, *p < 0.05) (Fig. 2B).

### Cultured platelets recirculate *in vivo* and undergo remodeling

**Figure 3:**
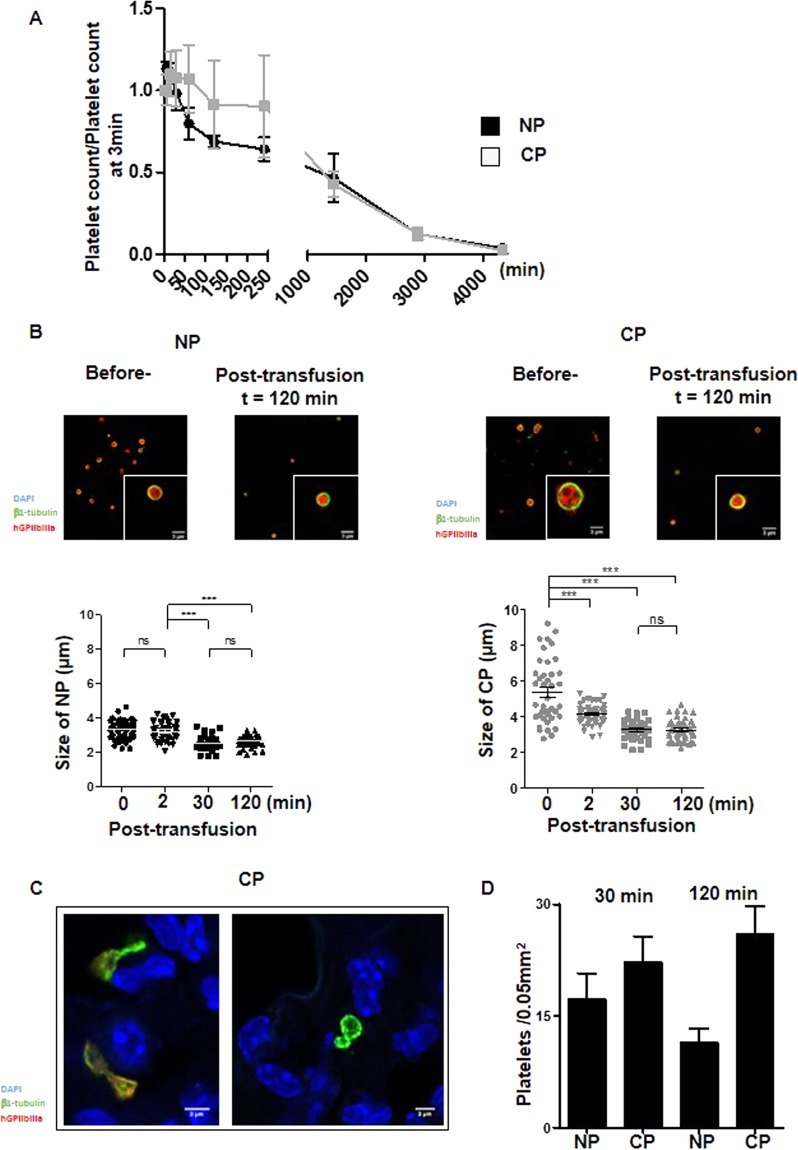
Functionality of cultured platelets. (**A)** *Platelet recirculation mouse model*. Cultured- or Native- platelets (1.10^8^) were transfused into NSG mice pretreated with clodronate liposomes. Blood samples were drawn prior to and at different time points over 72 h following transfusion. Human platelets were detected and counted by flow cytometry. Native platelets decreased linearly over time whereas Cultured platelets reached a plateau 15 min after injection and remained constant for at least 2 h, to progressively decrease and disappear completely after 3 days. Mean of 5 independent experiments. **(B)** *Platelet size*. Native platelets and Cultured platelets were examined by confocal microscopy prior to and 120 min after infusion into NSG mice. Washed platelets were cytospun, immobilized on poly-L-lysine and incubated with antibodies against β1-tubulin (green) and human GPIIb-IIIa (red). Scale bar = 1 µm (upper panels). Dot plots represent the diameter of Native platelets or Cultured platelets on confocal images at different time points following infusion of human platelets (lower panels). **(C)** Representative confocal images of lung cryosections from NSG mice infused with Cultured platelets. The lungs were removed 30 or 120 min after transfusion, embedded in a cryogenic gel and stained with antibodies against β1-tubulin (green) and GPIIb-IIIa (red). Cultured Platelets were more amenable to remodeling than Native platelets. **(D)** Bar graphs represent the quantification on confocal images of platelet remodeling (twisted microtubules) in the lungs of NSG mice infused with Native platelets or Cultured platelets. Values are the mean ± SEM in 3 separate experiments.

Two essential properties need to be considered for the use of cultured platelets in transfusion: their capacity to recirculate and their ability to sustain hemostasis. The first property was evaluated by infusing Cultured- or Native- platelets into macrophage-depleted immuno-deficient NSG mice and following the human platelet count in the circulation over time. Analysis of the curves revealed a maximal count at 15 min for either Cultured or Native platelets, with disappearance of all infused platelets by day 3 in both cases (Fig. 3A). While the 24 h half-life was similar for Cultured and Native platelets, the kinetics of clearance were different. Thus, following the peak at 15 min, a progressive and linear decrease in Native platelets was observed whereas numbers of Cultured Platelets remained constant for at least 2 h before beginning to decline.

One hypothesis to account for the plateau in Cultured Platelets count would be that these larger platelets remodel to give rise to smaller daughter platelets during recirculation, similarly as in the proplatelet fission process described by Thon *et al*.^19^. This hypothesis was tested by monitoring the platelet size, i.e. by measuring the diameter of the marginal band, at different time points after transfusion (Fig. 3B). Before transfusion, Cultured Platelets displayed a wide range of sizes, whereas after transfusion they became progressively homogenous and smaller to reach a size close to that of Native platelets (5.4 ± 0.3 µm before injection vs 3.2 ± 0.1 µm 120 min after injection, n = 3, ***p < 0.001). In comparison, the size of Native platelets decreased only marginally from 3.2 ± 0.1 to 2.5 ± 0.1 µm (n = 3, ***p < 0.001) (Fig. 3B). Since it is well documented that proplatelets can fragment and remodel in the pulmonary circulation^20^, we used confocal microscopy to examine the lungs of mice following infusion of human Cultured- or Native- platelets (Fig. 3C). Platelet elements with twisted microtubules, identified as β1 tubulin-positive events, were frequently observed after infusion of Cultured Platelets (26 ± 3.7% vs 11.4 ± 2% for Native platelets, n = 5, ***p < 0.001). Altogether, these results favor a mechanism where Cultured Platelets are remodeled during passage through the pulmonary circulation, contributing to an early stabilization of the platelet count (Fig. 3D).

### Cultured platelets limit blood loss

**Figure 4:**
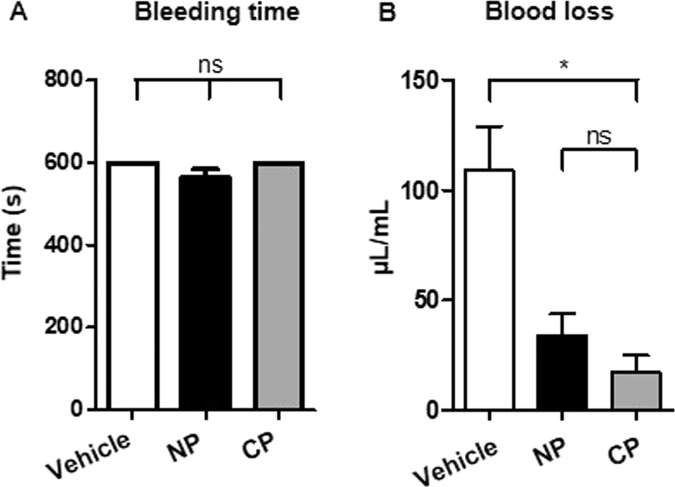
(**A**) Bleeding time. Profound thrombocytopenia was induced in NSG mice 24 h before the assay by injecting an anti-GPIbα mAb (RAM.6). On day 0, a tail bleeding assay was performed 10 min after retro-orbital injection of vehicle or 3.10^8^ Native platelets or Cultured platelets. **(B**) Blood loss was measured over a period of 10 min and values are the mean ± SEM in 3 separate experiments, *p < 0.05.

The second essential property we evaluated was the capacity of Cultured Platelets to support hemostasis. This was done by performing a tail bleeding assay in NSG mice which had been made severely thrombocytopenic (30.10^3^ plts/µL). Without transfusion of platelets, bleeding did not cease during a 10 min period of observation in this assay (Fig. 4A). Infusion of 3.10^8^ Cultured- or Native- platelets, which was expected to rescue only 15% of the normal platelet count in the mouse, was also insufficient to cause cessation of bleeding over 10 min (Fig. 4A). However, administration of Cultured platelets was sufficient to reduce blood loss with respect to the untreated control, as estimated from the amount of hemoglobin (Hb) in the blood collected (17.3 ± 7.7 vs 108.8 ± 2 µg/mL Hb, n = 3, **p < 0.01). This reduction was even more pronounced than after infusion of Native platelets (33.9 ± 9.9 µg/mL Hb) (Fig. 4B). Overall, these results indicated that Cultured platelets conserve hemostatic properties following transfusion.

## Discussion

In this study, we differentiated megakaryocytes from peripheral CD34^+^ cells in the presence of the chemical compound SR1. In this condition, MK are able to release more than 96% CD41-CD42b positive platelet-like elements following successive pipetting attesting the purity of the future transfused elements (data not shown). We established that cultured human platelets are functional, recirculate efficiently and limit blood loss in a thrombocytopenic NSG mouse model. These properties fulfill the essential prerequisites for the use of platelets generated *in vitro* in transfusion medicine. Moreover, these platelets were obtained without addition of serum or any animal components, which should allow the development of a platelet manufacturing protocol compliant with current good manufacturing practices.

We observed that Cultured platelets were larger than Native platelets, in agreement with the findings of other groups^21^. A large size is characteristic of newly formed or young circulating platelets. Accordingly, we observed that the cytoplasm of Cultured platelets was enriched in MVB and endoplasmic reticulum and contained large amounts of RNA, which are features of young platelets, usually encountered during reactive thrombocytosis after induction of severe thrombocytopenia^15^. In contrast, steady state circulating platelets display an age continuum, from young to old. The transfusion of only young platelets, as obtained in culture, could represent a potential benefit as they would provide better hemostatic protection than platelets from donors.

As reported in many other studies, Cultured platelets appeared to be slightly pre-activated, as illustrated by their exposure of P-selectin and binding of fibrinogen in the absence of added agonist (Suppl. Fig. 3B,C). This activation can be explained by the absence *in vitro* of endothelial cell products such as NO and PGI_2_, which are normally present *in vivo* and reduce platelet activation. Prior to transfusion, Cultured platelets aggregated in response to ADP and TRAP. Since they lack fibrinogen in their α-granules, thrombin was unable to directly induce platelet aggregation (Suppl. Fig. 1A,B). Culture of platelets in the presence of fibrinogen could be envisaged to solve this problem, but might not be necessary since endocytosis from plasma appears to occur soon after transfusion of Cultured platelets.

When assessing their capacity to recirculate, the half-life of cultured platelets was found to be equivalent to that of native ones. We estimated that the proportion of platelets cleared immediately after transfusion (within 3 min) represented around 20%, in Cultured- or Native-platelets further indicating that Cultured platelets acted as *bona fide* platelets (Suppl Fig. 2B,C). Following transfusion, Cultured platelets progressively lost their TO staining (Suppl. Fig. 3A) and became smaller to reach the size of Native platelets (Fig. 3B). This can be explained by their remodeling attested by the presence of numerous platelets displaying a twisted morphology in the lungs of mice transfused with Cultured platelets (Fig. 3C,D). These observations recall those of J. Italiano’s group concerning the conversion of preplatelets, anucleate discoid particles 2–10 µm in diameter, into barbell-shaped proplatelets through microtubule-driven twisting forces^19^. It may be hypothesized that, constrained by the pulmonary microcirculation, Cultured Platelets remodel and divide to form larger numbers of daughter platelets, explaining the initial maintenance of a constant platelet count. It is noteworthy that following transfusion, circulating Cultured platelets no longer exhibited activation and were responsive to thrombin stimulation (Suppl. Fig. 3B,C). Whether pre-activated platelets are eliminated or whether they are passivated through contact with endothelial cells in the blood vessels is at present not known.

An alternative to the transfusion of cultured platelets has been proposed in the form of the infusion of cultured MK into mice. The platelets generated *in vivo* from these cells were mainly released from MK trapped within the pulmonary circulation. They displayed a normal size and as in the case of Cultured platelets produced *ex vivo*, an almost normal half-life in the blood stream. However, it is difficult to compare their functional properties as they could not be separated from the native platelets of the recipient mice^22^. In addition, a possible complication due to the accumulation of infused MK nuclei in the lungs would be avoided in Cultured platelets.

Additionally, of all the criteria that can be assessed, Cultured platelet satisfied the most reliable for a clinical application, i.e., these platelets limited blood loss and were thus capable of ensuring primary hemostasis upon transfusion. According to this study, it may be expected that Cultured platelets will fully protect against bleeding when scale-up production allows the transfusion of larger numbers of platelets into NSG mice.

To conclude, we could demonstrate here that CD34^+^-derived SR1- Cultured platelets exhibit properties of *bona fide* human platelets. In addition, with the technological breakthroughs made in the last few years to produce large amounts of platelets with a high quality, the concept of cultured platelets as a transfusion alternative thereby becomes realistic. Nevertheless, several obstacles remain to be overcome before it might be possible to introduce the routine use of such innovative blood components.

## Supplementary information

supplemental figures.
