# Supplementary material for: Functional properties of human platelets derived in vitro from CD34+ cells
Source: Sci Rep. 2020 Jan 22;10:914. doi: 10.1038/s41598-020-57754-9 (PMC6976668; doi:10.1038/s41598-020-57754-9)
Supplement: Supplementary file 1 — supplemental figures. [file 41598_2020_57754_MOESM1_ESM.pdf]

Functional properties of human platelets derived *in vitro* from CD34$^+$ cells

Do Sacramento V, Mallo l, Freund M, Eckly A, Hechler B, Mangin P, Lanza F, Gachet C, Strassel C

Université de Strasbourg, INSERM, EFS-Grand Est, BPPS UMR_S1225, FMTS, F-67000 Strasbourg, France

Correspondence to :

Catherine Strassel, UMR_S1255, EFS-Grand Est, site de Strasbourg, 10 rue Spielmann, F-67065 Strasbourg Cedex, France

Tel: (33) 388 21 25 25; Fax: (33) 388 21 25 21; E-mail: catherine.strassel@efs.sante.fr

Supplemental Methods

Platelet aggregation. Aggregation was measured at 37°C by a standard turbidimetric method in an APACT 4004 aggregometer (ELITech Group, Puteaux, France) [14]. Briefly, a 135 µL aliquot of platelet suspension containing 20.10^6 CP or NP was stirred at 1,100 rpm and activated by addition 0.1 U/mL thrombin, in a final volume of 150 µL. The extent of aggregation was estimated with APACT LPC software.

Fibrinogen content. After fixation in paraformaldehyde, CP and NP were cytospun, immobilized on poly-L-lysin, permeabilized with 0.1% Triton X-100 in PBS and incubated sequentially for 30 min, with antibodies against fibrinogen (green) and GPIbβ (RAM.1, red) and with DAPI (blue) for nuclear staining.

Activation assay. Washed NP or CP were stimulated at different time points after infusion into NSG mice, with thrombin (1 U/mL) in the presence of FITC-anti-P-selectin antibody (25 µg/ml), mixed and at 10 min fixed with PBS-20 mg/ml PFA for 20 minutes (min). Platelets were pelleted by centrifugation at 1,000 x g for 2 min and resuspended in 500 µl phosphate-buffered saline (PBS). The fluorescence intensity was measured using a Fortessa-X20 flow cytometer (BD, Biosciences).

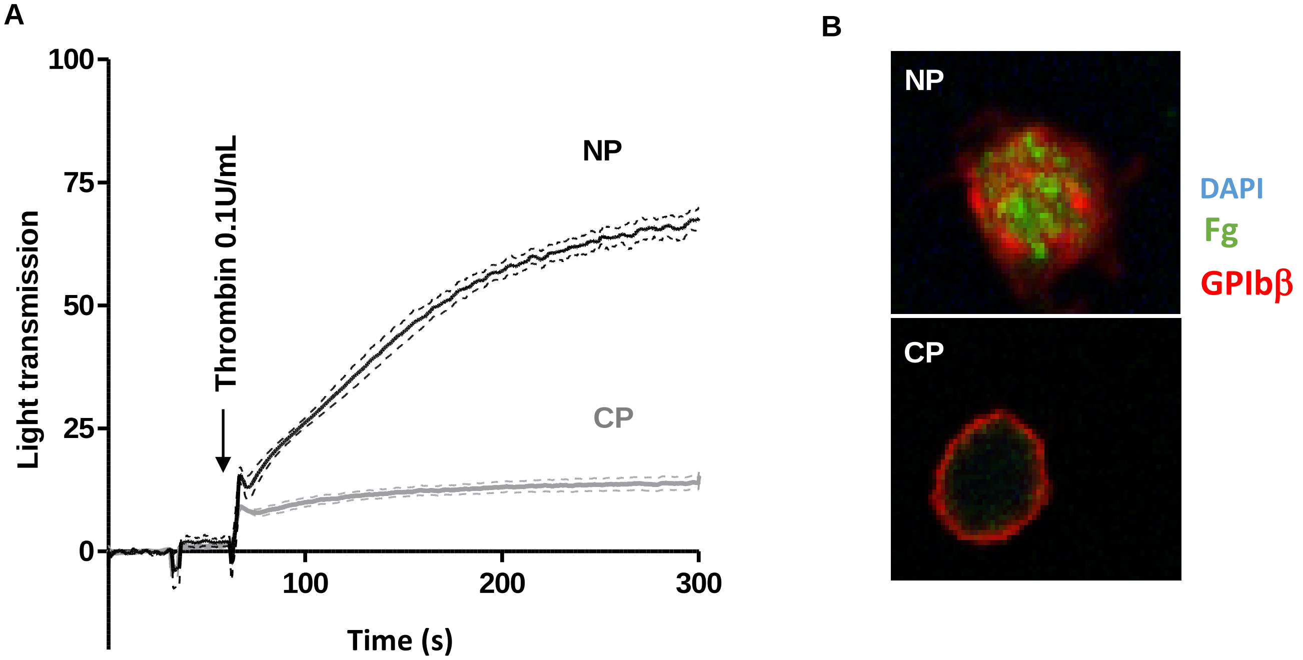

Supplemental Figure 2: (A) *Aggregation assay*. Washed human platelets were stimulated with thrombin (0.1 U/mL) in the absence of fibrinogen (CP vs NP: 15.1±1.1 % vs 65.7±1.4 %, n=3). **(B)** *Fibrinogen content*. CP and NP were cytospun, immobilized on poly-L-lysine and incubated with antibodies against fibrinogen (green) and GPIbβ (RAM.1, red) and with DAPI (blue) for nuclear staining. Images were obtained by confocal microscopy, scale bar = 1 µm.

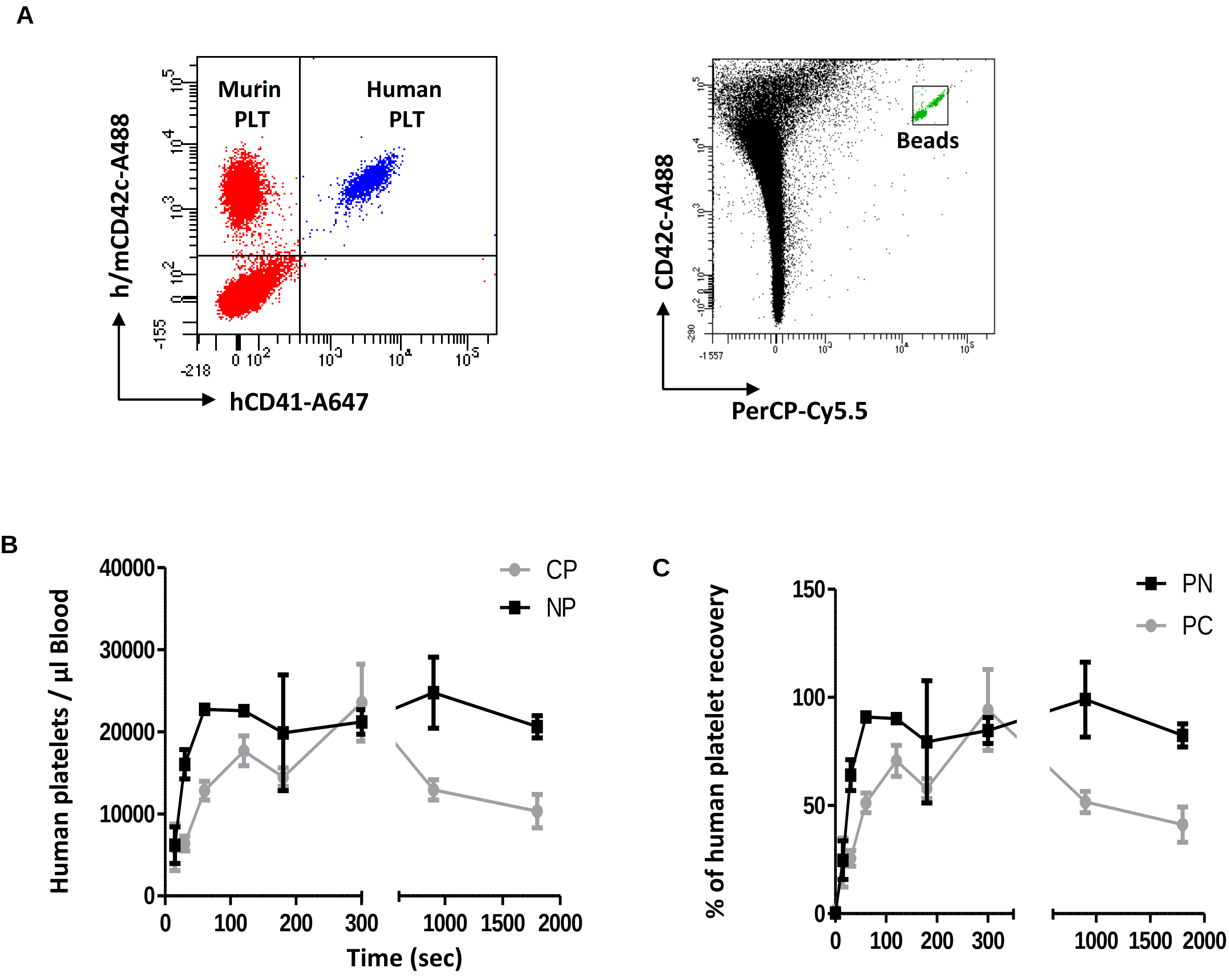

Suppl. Fig. 2: Short time murine circulation model. (A) Flow cytometry analysis. Blood samples were drawn prior to and at different time points over 30min following transfusion in a Trucount™ Tubes (BD bioscience) and labeled with specific antibodies. Counting beads are gated and acquisition of events was stopped when 5000 bead events were acquired. (B) The graph represents the number of human platelets circulating in mouse/µL of blood. Values are the mean ± SEM in 2 for Native platelets and 3 for Cultured platelets separate experiments. (C) The graph represents the % of human platelet recovery, values are the mean ± SEM in 3 separate experiments.

Supplemental Figure 2.

A

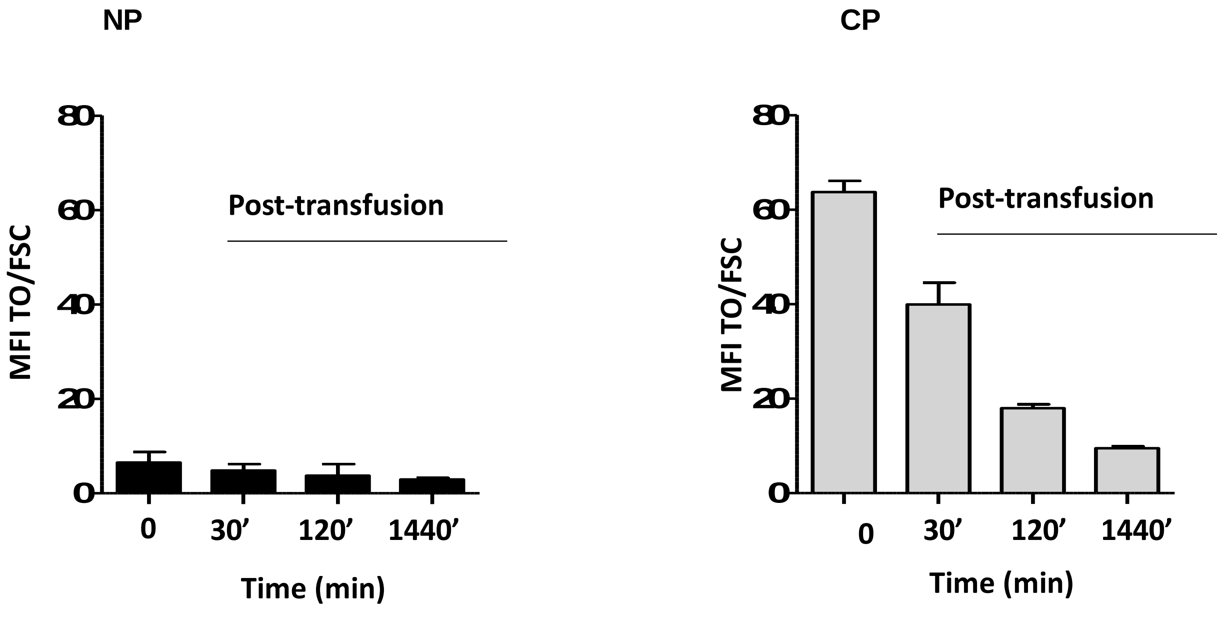

Supplemental Figure 3: Characterization of cultured platelets after recirculation. (A) *Reticulated platelets*. Bar graphs represent the relationship between the thiazol orange (TO) staining and size (FSC) of NP or CP before and at different time points (30, 120 and 1440 min) following infusion into NSG mice. **(B-C)** *Activation assay.* Bar graphs represent the mean fluorescence intensity of (B) P-selectin expression and (C) fibrinogen binding, in the presence or absence of thrombin, in NP or CP before injection (BI) and at different time points (60 and 120 min) after infusion into NSG mice.

Supplemental Figure 3.

B

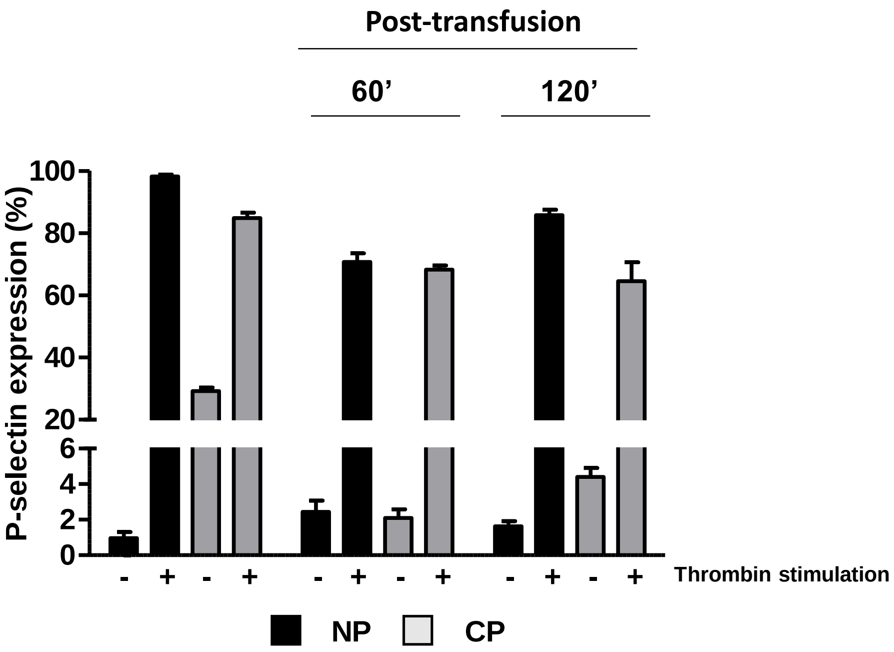

C

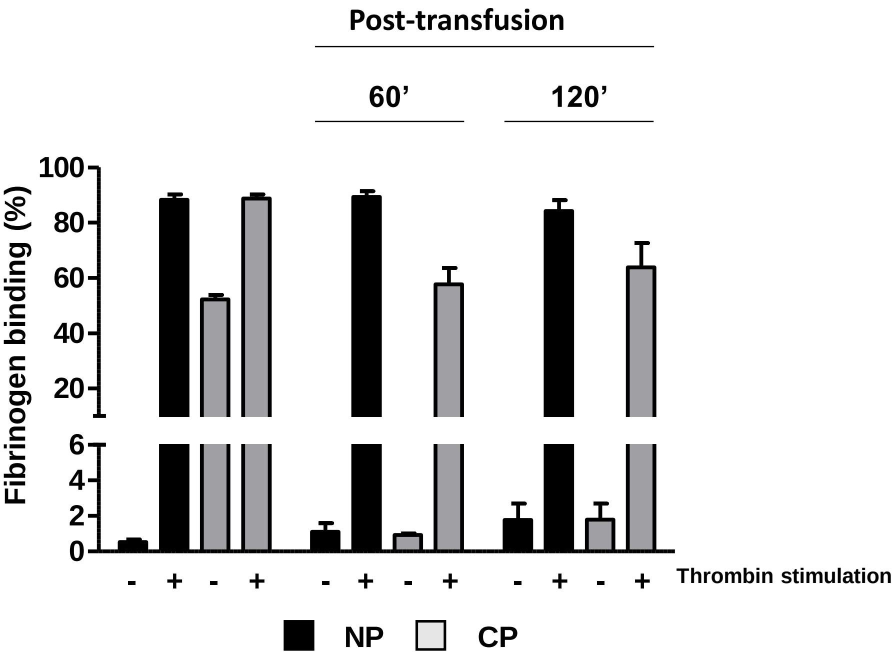

Supplemental Figure 3.
